# Disability as Diversity: A Guidebook for Inclusion in Medicine, Nursing, and the Health Professions

**DOI:** 10.5195/jmla.2021.1242

**Published:** 2021-07-01

**Authors:** Elizabeth Moreton

**Affiliations:** 1 emoreton@email.unc.edu, Health Sciences Library, University of North Carolina at Chapel Hill, Chapel Hill, NC

*Disability as Diversity: A Guidebook for Inclusion in Medicine, Nursing, and the Health Professions* is a basic introduction for those who want to learn about accessibility issues in health sciences higher education programs. Targeted toward instructors, this book covers barriers to learning for students with disabilities, the process of seeking accommodations, some introductory classroom or clinical accommodations, legal aspects or standards of disability, and examples of previous legal cases. Throughout the book, the authors highlight how in many cases, students are responsible for seeking accommodations or navigating legal processes in the college setting without any advice or assistance.

While this book does provide a good introduction to issues for students with disabilities, it may not satisfy those looking for practical advice on how to best accommodate students in the classroom, lab, or clinical setting, nor does it provide new information for those who have already learned the basics of accessibility in higher education. Those readers may instead want to read Meeks's earlier book, *The Guide to Assisting Students With Disabilities: Equal Access in Health Science and Professional Education* [1], which provides more practical solutions in accessibility for students with disabilities. Many of the classroom accommodations listed in *Disability as Diversity* have been used in the college setting for at least ten years. *Disability as Diversity* provides a few novel solutions, such as using simulation to determine what accommodations would be necessary for a student in a health program to work or learn in a clinical setting (p. 254–6).

As a librarian with a moderate amount of background in accessibility issues in college education, I have some concerns about how the authors approached the idea of disability and student accommodations in this book. The writing in some parts of this book can be problematic, and at times the authors generalize people with disabilities in a somewhat stereotypical way by suggesting functional limitations associated with different disabilities can be solved by the same accommodations (p. 217). Some solutions are a bit heavy-handed, such as suggesting students with chronic illness–related disabilities avoid flares or wake up earlier to take care of themselves so they can arrive on time (p. 228) or suggesting another staff person take over the responsibilities of the student with disabilities if they are too slow getting to a “Code Blue” (p. 245), rather than providing other more equivalent alternatives for the student. In my opinion, readers should view this book as introductory only and continue learning about this topic from a variety of perspectives.
